# Laparoscopic versus Open Complete Mesocolic Excision for Right Colon Cancer

**DOI:** 10.1155/2021/8859879

**Published:** 2021-02-02

**Authors:** Ali Zedan, Essam Elshiekh, Mohamed I Omar, Mohamad Raafat, Salah M. Khallaf, Haisam Atta, Marwa T. Hussien

**Affiliations:** ^1^Department of Surgical Oncology, South Egypt Cancer Institute, Assiut University, Asyut, Egypt; ^2^Department of Surgical Oncology, Tanta Cancer Center, Tanta, Egypt; ^3^Department of General Surgery, Faculty of Medicine, Assiut University, Asyut, Egypt; ^4^Department of Medical Oncology, South Egypt Cancer Institute, Assiut University, Asyut, Egypt; ^5^Department of Diagnostic Radiology, South Egypt Cancer Institute, Assiut University, Asyut, Egypt; ^6^Department of Oncologic Pathology, South Egypt Cancer Institute, Assiut University, Asyut, Egypt

## Abstract

**Results:**

The mean operative time was significantly longer in the LCME group than that in the OCME group with less mean intraoperative blood loss. Conversion was required in 4 patients (8.3%) in the LCME group. The use of laparoscopy increased the number of harvested lymph nodes compared to the open approach (39.81 ± 16.74 vs. 32.65 ± 12.28, respectively, *P*=0.010). The laparoscopic approach was associated with a shorter time interval to first flatus as well as shorter time interval to liquid and normal diet after surgery. The postoperative hospital stay was significantly shorter in the LCME group. The complication rate was slightly lower in the LCME (14.7%) than in the OCME group (27.2%) (*P*=0.252). The 3-year OS in the LCME group was similar to that in OCME (78.2% vs. 63.2%, respectively, *P* value = 0.423). The three-year DFS in the laparoscopic group was higher (74.5%) than the open group (60.0%), but did not reach statistical significance (*P* value = 0.266).

**Conclusions:**

In conclusion, laparoscopic CME right hemicolectomy is a technically feasible and safe procedure if surgeon expertise is present. LCME has long-term oncologic outcomes (recurrence and survival) comparable to open surgery for management of patients with stage II or III colon cancer.

## 1. Introduction

In 2009, Dr. Hohenberger first proposed the concept of complete mesocolic excision (CME) for colon cancer surgery [1] according to the concept of total mesorectal excision (TME) for rectal cancer. The technique of CME relies on three key components: (I) sharp dissection in the embryologic plane between the parietal fascia and visceral (mesenteric) fascia to remove mesentery together with its lymphatic drainage as an intact envelope [2]; the principle behind this technique is to avoid any inadvertent exfoliation of the tumor cells from mesentery into the peritoneal cavity; (II) proximal ligation of feeding vessels at their origin to remove apical lymph nodes; and (III) resection of a sufficient length of bowel to remove potentially involved lymph nodes in a longitudinal direction [3].

After postulation of CME technique, CME with central vascular ligation (CVL) has been applied by many European centers for colon cancer [2]. Also, in Japan, Korea, and a number of other Asian countries, a D3 lymphadenectomy technique, which has a concept close to CME, has been gaining popularity [4, 5]. CME and D3 lymphadenectomy are associated with higher reported survival rates than conventional colon resection surgery [3]. These results made many surgeons consider CME and D3 lymphadenectomy techniques as the standard of care for clinical stage II and III colon cancer surgery.

After introduction of the use of laparoscopic approach for colorectal surgery, several randomized trials [6–8] and systematic reviews [9, 10] have shown that the laparoscopic approach for colon cancer surgery is associated with faster recovery and less morbidity as compared to the standard open approach without affecting oncologic outcomes [11–13]. Recently, several studies have discussed the feasibility of laparoscopic CME, primarily for right colon cancer with promising results [9, 14–17].

The aim of the present study is to compare the laparoscopic approach for CME with the open approach in right colon cancer treatment with regard to the feasibility, safety, and perioperative and oncologic outcomes.

### 1.1. Patients and Methods

This is a prospective study which included all patients that underwent radical right hemicolectomy for pathologic confirmed stage II or stage III tumor with CME at South Egypt Cancer Institute, Assiut University, from January 2012 to December 2019. The exclusion criteria included colon cancer with stage IV disease, synchronous or previous malignancies, and those with extracolonic invasion (T4b). Moreover, non-malignant cases and emergency cases with bowel obstruction or intestinal perforation were excluded from the study. The study was approved by the institutional review board (IRB). All enrolled patients were divided into laparoscopic colectomy (LCME) group (*n* = 48) or open colectomy (OCME) group (*n* = 48) according to the surgical approach. The choice of the type of operation was largely depending on the tumor characteristics and surgeon preference. In early cases, the learning curve was a bit slow which made the number of laparoscopic cases fewer and restricted to less technically demanding cases.

Preoperative evaluation consisted of history taking, physical examination, basic laboratory studies including serum CEA, preoperative full colonoscopy, abdominal ultrasound, CT abdomen and pelvis, chest x-ray/CT chest, and selective PET/CT.

Surgical outcome parameters included incision length, operative time, blood loss, conversion rate, postoperative pain score, postoperative first passage of flatus, duration of hospital stay, and postoperative morbidity and mortality within 30 days after surgery.

Oncologic outcome parameters, including tumor size, distal, proximal, and circumferential resection margins, number of lymph nodes retrieved, and TNM classification, were collected.

Follow-up data, including use of chemotherapy, local recurrence rate, distant metastasis rate, and short-term survival data (including overall survival and recurrence-free survival rates), were collected.

### 1.2. Surgical Technique

#### 1.2.1. Open Approach

**Figure 1:**
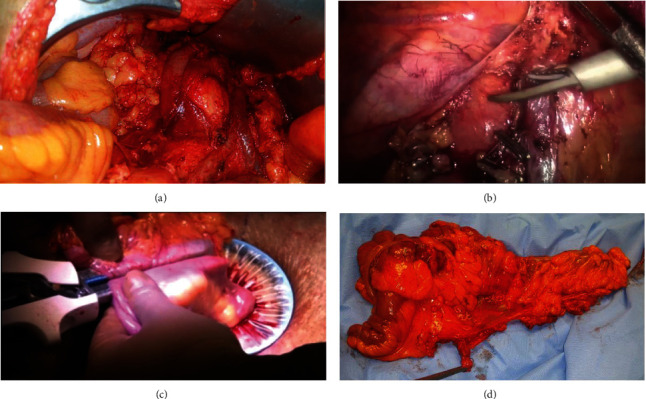
Surgical technique and pathologic examination. (a) Open CME with division of feeding vessels at their origin. (b) Laparoscopic CME showing clipped ileocolic vessels at their origin from superior mesenteric vessels. (c) Side-to-side stapled ileocolic anastomosis after LCME right hemicolectomy with use of wound protector. (d) CME specimen for gross pathologic assessment before fixation.

Lateral-to-medial approach, starting via incision of Toldt line, is used for complete mobilization of the colon. The visceral and parietal fasciae are separated by sharp dissection to ensure an intact mesocolon. The feeding arteries are transected close to their origin from the superior mesenteric artery at the left side of SMV (central vascular tie). For cecal, ascending colon, and hepatic flexure tumor, right hemicolectomy is performed with division of the colon at mid-transverse and division of the ileum 10 cm of the terminal ileum. The ileocolic, right colic (if present), and right branch of middle colic vessels are divided. For tumors distal to hepatic flexure, extended right hemicolectomy is performed with resection of proximal 2/3 of the transverse colon and division of middle colic vessels at their origin (Figure 1(a)). For tumors of hepatic flexure and proximal transverse colon, a part of greater omentum is removed en bloc with the specimen. An end-to-end or end-to-side ileocolic anastomosis is performed using a hand-sewn technique with 3-0 Vicryl suture.

#### 1.2.2. Laparoscopic Approach

Medial-to-lateral approach is used in all cases. Firstly, the mesentery at the junction of the terminal ileum and cecum is grabbed and pulled to the right lower quadrant to identify the ileocolic pedicle. The peritoneum on the caudal aspect is incised parallel to the arc of the ileocolic vessels and dissection proceeds to enter into the retroperitoneal plane. Then, sharp dissection proceeds from the medial to lateral and in caudal-cephalic direction to separate the posterior layer of the mesocolon from the parietal fascia, exposing and protecting right gonadal vessels, ureter, duodenum, and head of the pancreas. This is followed by the dissections of gastrocolic ligament, right side of greater omentum, and lateral peritoneum of the colon. Division of vessels is similar to that discussed in the open approach at their origin from superior mesenteric vessels (central vascular tie) (Figure 1(b)). The pedicle vessels are ligated with hemoclips and divided using bipolar vessel-sealing devices. Bowel resection is similar to open approach.

The specimen was extracted from a small midline incision (about 5 cm) proximal to the umbilicus. The use of a wound protector for extraction is recommended to minimize the risk of a wound contamination and tumor spillage. An extracorporeal end-to-end or end-to-side ileocolic anastomosis is performed using hand-sewn or stapling techniques (Figure 1(c)).

### 1.3. Pathological Examination

Gross examination of the CME-colectomy specimen is done firstly to check for quality of resection (Figure 1(d)). Generally, 6 items are assessed: (1) morphologic assessment of the plane of dissection, (2) length of colon resected, (3) length of high tie vascular ligation of the mesenteric artery to the colon, and (4) the mesocolic area, (5) the shortest distance from the vascular high ligation point to the tumor and intestine, and (6) number of lymph nodes harvested. The American Society of Clinical Oncology (ASCO) recommends that >12 lymph nodes are needed for accurate staging. Afterwards, the specimen was formalin-fixed paraffin-embedded (FFPE), forming tissue blocks. Blocks were cut into 5 *μ*m thick and mounted on glass slides. Slides were stained with hematoxylin and eosin stain for light microscopy evaluation.

If there are difficulties in determining the tumor depth in T4 lesions, we perform double staining using Victoria blue and hematoxylin-eosin to identify the serosa of the colon.

#### 1.3.1. Follow-Up and Surveillance

Physical examination is every 3 months for the first 2 years and every 6 months for the next first 2 years and then annually for the following years. A chest X-ray and abdominopelvic computed tomography (CT) scan and serum carcinoembryonic antigen (CEA) assay are performed every 6 months for the first 2 years and yearly thereafter. Colonoscopy was performed at the first anniversary and then after 3 years.

#### 1.3.2. Statistical Methods

SPSS version 23.0 was used for data management. Mean and standard deviation described quantitative data and count with percentages for qualitative data. Quantitative data were compared using *t*-test and qualitative data using Chi-square/Fisher exact tests. Overall survival was calculated from the date of pathologic diagnostic confirmation to the date of death or last follow-up, and disease-free survival was calculated from the date of curative surgery up to the first evidence of either local recurrence or distant metastasis or both. Kaplan–Meier methods were used to estimate survival and log-rank test to compare curves with *P* value always 2-tailed and significant at the 0.05 level.

## 2. Results

This study included a total of 96 patients (54 males and 42 females), divided into laparoscopic the colectomy (LCME) group (*n* = 48) or open colectomy (OCME) group (*n* = 48) according to the surgical approach.

### 2.1. The Patients' Demographic Characteristics

There were no statistically significant differences between the 2 groups as regards age, gender, or comorbidities. Mean age was 61.56 ± 13.52 in OCME and 59.53 ± 10.92 in LCME (*P*=0.642).

### 2.2. Pathologic Parameters

The differences as regards tumor location, tumor differentiation, and TNM stage were statistically insignificant between the two groups. The most common tumor location in both groups was the ascending colon (67 patients). Moderately differentiated tumors were more prevalent in both groups. Stage III was the final pathologic stage in 55 patients vs. 41 cases with stage II (Table 1).

### 2.3. Operative Data and Technical Aspect according to the Surgical Approach

The mean length of specimen in the open group was about 27.3 ± 3.2 cm which is significantly longer than laparoscopic group (20.1 ± 1.97 cm; *P* < 0.001). Mean tumor size was 5.6 ± 0.9 in OCME vs. 4.5 ± 0.7 in LCME with *P* < 0.001. Similarly, the area of mesentery was higher in OCME than LCME (15.36 mm2; vs. 14.22 mm2, respectively) with *P* < 0.001. Sufficient proximal and distal resection margins were obtained in both groups. Patients from the laparoscopic group had a shorter incision, with mean length 6.10 vs. 18.79 cm. The mean operative time was significantly longer in the LCME group than that in the OCME group with less mean intraoperative blood loss. There was a greater distance between the tumor and high tie in a LCME group with greater distance between nearest bowel wall and high tie. The use of laparoscopy increased the number of harvested lymph nodes compared to the open approach (39.81 ± 16.74 vs. 32.65 ± 12.28, respectively, *P*=0.010). There was no difference in the achievement of an intact mesocolic plane in the laparoscopic group vs. the open group (77.1%% vs. 83.3% *P*=0.609) (Table 2).

### 2.4. Postoperative Data according to the Surgical Approach

Postoperatively, patients in LCME group had lower pain scores than OCME group (3.25 ± 0.76 vs. 5.31 ± 0.88, *P* < 0.001). The laparoscopic approach was associated with a shorter time interval to first flatus (56.77 hs vs. 100.48 hs, *P* < 0.001). The LCME patients had a shorter time interval to liquid diet as well as a shorter time interval to normal diet after surgery. The postoperative hospital stay was significantly shorter in the LCME group (9.13 d) than in the OCME group (13.04 d), *P* < 0.001 (Table 3).

### 2.5. Postoperative Morbidity

The complication rate was lower in the LCME (14.7%) than the OCME group (27.2%) (*P*=0.252). There was also no significant difference between both groups in incidence of wound infection, lymphatic leakage, anastomotic leakage, pulmonary infection, ileus, deep venous thrombosis, and 30-day mortality. Conversion rate was required in 4 patients (8.3%) in the LCME group (Table 4). The reasons for conversion were technically difficult dissection in two patients, severe adhesion in one patient, and uncontrolled bleeding in one patient. One patient in the OCME group died due to pulmonary embolism while one patient in the LCME group died due to sepsis after anastomotic leakage. The two patients in OCME group who developed anastomotic leakage were managed by peritoneal lavage and exteriorization of ileocolic anastomosis.

### 2.6. Recurrence Rate

Local recurrence occurred in two patients in each group. Distant metastasis occurred in 7 patients, with no significant differences between the two groups (Table 5). Four cases metastasized to the liver, 2 cases to the lung, and one patient developed peritoneal carcinomatosis.

### 2.7. Survival Data

**Figure 2:**
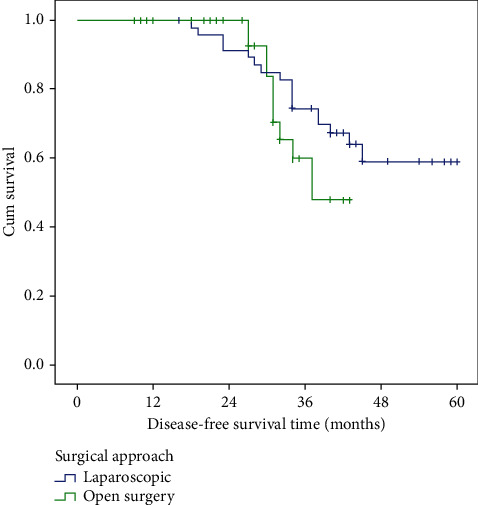
Kaplan–Meier survival function curve. It shows comparable survival rates between the 2 study groups regarding relapse-free survival rates.

The 3-year overall survival (OS) in the whole group was 74.0% ± 5.3. The 3-year OS in the LCME group was similar to that in OCME (78.2% vs. 63.2%, respectively, *P* value = 0.423). Disease-free survival (DFS) at 3 years in all cases was 70.5% ± 5.5. The three-year DFS in the laparoscopic group was higher (74.5%) than the open group (60.0%), but did not reach statistical significance (*P* value = 0.266) (Figure 2).

## 3. Discussion

After postulation of CME by Hohenberger et al. [1], this technique has been widely practiced in many high-volume centers for colorectal cancer surgery. Most colorectal surgeons believe that CME colectomies are superior to “traditional” ones in terms of local recurrence and cancer-related survival. The interest in the last years focuses on decreasing the perioperative morbidity through the use of minimally invasive approaches for patient undergoing colon cancer surgery. Performing CME technique via laparoscopy aimed to reach maximum oncologic quality of cancer surgery with least perioperative morbidity. Although the laparoscopic right hemicolectomy is widely performed globally, the feasibility and safety of laparoscopic CME have only recently been demonstrated in a few centers [14, 15, 18].

Some classic operative advantages of laparoscopic CME compared with open surgery include less blood loss and fewer transfusions [7, 8, 19, 20]. Similarly, in our work, although the mean operative time was significantly longer in the LCME group than that in the OCME group, there was less mean intraoperative blood loss.

In this study, recovery of gastrointestinal function after surgery in the laparoscopic approach was faster than OCME with a shorter time interval to first flatus, liquid diet, and normal diet and less incidence of ileus. This is consistent with the solid concept about the prominent advantages of laparoscopic surgery in terms of better GIT recovery (which has been reported in many large randomized trials as COST, CLASSIC, COLOR I, and ALCCaS trials) [8, 11, 19, 20]. In addition, many authors who compared LCME with OCME reported similar findings [14, 17]. In a large meta-analysis which investigated the outcome of LCME in 11 studies, they concluded that in 4 studies a shorter time interval to first flatus was reported with laparoscopic approach, with a mean difference of 0.9 days. Also, the time to liquid diet (reported by 5 studies) was shorter for the LCME patients, with a mean difference of 1.84 days [16].

The overall complication rate was slightly lower in the LCME group (14.7%) than the OCME group (27.2%) in our work (*P*=0.252). This was close to the data in a retrospective study reported by Wang et al., who showed that the short-term complication rate within 30 days was 16.3% [21]. Also, Huang et al. noted that, in a retrospective analysis of 102 patients, the complications rates of LCME (4%) and OCME (12%) were comparable [22]. Anastomotic leakage, pulmonary infection, and deep venous thrombosis were higher in the open group than laparoscopic group, despite not reaching statistical significance. There was no significant difference between both groups in incidence of wound infection and lymphatic leakage. These data are supported by reports of many authors who showed comparable complication rates between open and laparoscopic approaches in CME technique [14, 17]. Fortunately, major operative complications, e.g., duodenal injury, mesenteric vessel injury, or ureteric injury, did not occur in our study. We concluded that laparoscopic approach, if not decreasing morbidity, will have no further hazards to the patients.

The postoperative hospital stay in our study was significantly shorter in the LCME group (9.13 d) than in the OCME group (13.04 d), *P* < 0.001. This can be explained by early GIT recovery. An analysis of the short-term outcomes of colon and rectal laparoscopic resections revealed shorter hospitalization time (5–10 vs. 6–11 d, *P*=0.0011) for laparoscopic procedures as in the COST, CLASSIC, COLOR I, and ALCCaS trials [8, 11, 19, 20]. Huang et al. showed that length of hospital stay was significantly shorter in the LCME group as compared to OCME group (11 ± 4 d vs. 14 ± 6 d).

The area of mesentery was higher in OCME than LCME (15.36 mm^2^ vs. 14.22 mm^2^, respectively) with *P* < 0.001. In their meta-analysis of relevant studies, Negoi et al. reported the surface of the resected mesocolon to be larger in the LCME group (MD = 11.75 cm^2^, *P* < 0.001) [16].

Our data showed a greater distance between the tumor and high tie in LCME group compared to OCME (140.42 ± 16.78 vs. 124.44 ± 18.61, *P* < 0.001) as well as a greater distance between the nearest bowel wall and high tie (110.71 ± 12.36 vs. 79.96 ± 8.84 *P* < 0.001). On the other hand, Gouvas and his colleague showed that distal ascending hepatic flexure, proximal transverse patients operated on by the open approach displayed significantly longer distances of tumor to the high tie (open 11.67 cm vs. laparoscopic 8.72 cm, *P*=0.049) and of the bowel wall to the high tie (open 9.11 cm vs. laparoscopic 6.5 cm, *P*=0.015). However, all other groups (cecum, proximal ascending colon, and left colon) showed comparable distance in terms of high tie to tumor and high tie to nearest bowel wall between laparoscopic and open groups [15].

In our work, we noted that the use of laparoscopy increased number of harvested lymph nodes compared to open approach (39.81 ± 16.74 vs. 32.65 ± 12.28, respectively, *P*=0.010). This is in disagreement with many authors who showed that the numbers of harvested lymph nodes between the two groups were comparable [14, 17]. In addition, in a large meta-analysis that included 11 studies, 10 of them reported the number of retrieved lymph nodes for 1376 LCME patients and 1271 OCME patients. They found no statistically significant mean difference between LCME and OCME (MD = ‒1.06, 95% CI: ‒3.65 to 1.53, *P*=0.42). These findings held true even after subgroup analysis according to the number of included patients (less or more than 100 patients in each group) and the geographical location of the study (Europe and Asia) [16].

In this study, conversion was required in 4 patients in the LCME group (8.3%). We regard this highly accepted as conversion rates in randomized controlled trials comparing laparoscopic and open colectomies ranged from 11% to 25% and the main cause of conversion in laparoscopic colectomy for cancer was local tumor progression [7, 8, 23].

Local recurrence occurred in two patients in each group while distant metastasis occurred in seven patients, with no significant differences between the 2 groups. Port site metastasis did not occur in our work. Sung et al. showed similar data with comparable overall rates of recurrence among groups (LCME 12.9% vs. OCME 20.0%, *P*=0.215) [14].

Qin-Song et al. showed that the 2 groups of patients were also similar in terms of local recurrence rate (LCME 1.3% vs. OCME 1.4%) [17]. In a large meta-analysis, 5 studies addressed the local recurrence rates in 1233 patients where there were no statistically significant differences between LCME and OCME (OR = 0.67, 95% CI: 0.38 to 1.17, *P*=0.16). On the other hand, distant recurrence rate was addressed by four studies, with no statistically significant differences between the two groups (OR = 0.98, 95% CI: 0.61 to 1.58, *P*=0.94) [16].

We found, in this study, comparable 3-year OS and DFS among both groups. The 3-year OS rate was 78.2% ± 6.1 in the laparoscopic group as compared to 63.2% ± 11.0 in open surgery, *P* value = 0.423. The 3-year DFS in the laparoscopic group was 74.5% ± 6.4 and in open surgery was 60.0 ± 10.6, *P* value = 0.266. In Negoi et al.'s meta-analysis, the 3-year OS was reported by four studies, including 1010 patients. The laparoscopic approach was associated with a statistically significant better 3-year OS, with an OR of 2.02 (95% CI: 1.31 to 3.12, *P*=0.001) [16]. In Qin-Song et al.'s study, during the follow-up period (median 20.1 ± 4.6 months), the laparoscopic and open groups were similar in terms of local recurrence rate (1.3% vs. 1.4%), distant metastasis rate (1.3% vs. 1.4%), and short-term survival rate (79.5% vs. 77.8%). These data are close to results of our study [17]. Sung et al. reported median follow-up period of 58 months in the LCME group and 61 months in the OCME group. The 5-year OS rates of the LCME and OCME groups were 77.8 and 90.3% (*P*=0.028), respectively, whereas the 5-year DFS rates were 71.8 and 83.3% (*P*=0.578), respectively [14].

## 4. Conclusions

In conclusion, laparoscopic CME right hemicolectomy is a technically feasible and safe procedure if surgeon expertise is present. Despite the longer operation time, LCME has better short-term clinical outcomes (less operative blood loss, less postoperative pain, faster recovery of gastrointestinal function, shorter hospitalization time, and higher lymph node yield). Moreover, LCME has long-term oncologic outcomes (recurrence and survival) comparable to open surgery for management of patients with stage II or III colon cancer.

## Figures and Tables

**Table 1:** Tumor site, pathology, TNM stage, and comorbidities.

		Surgical approach				*P* value^*∗*^
		Open surgery		Laparoscopic		
		Count	%	Count	%	
Tumor locations	Ascending colon	33	68.8	34	70.8	0.188
	Cecum	8	16.7	12	25.0	
	Hepatic flexure	7	14.6	2	4.2	

Pathological type	Well differentiated	6	12.5	9	18.8	0.451
	Moderately differentiated	29	60.4	31	64.6	
	Poorly differentiated	13	27.1	8	16.7	

TNM stage	II	19	39.6	22	45.8	0.680
	III	29	60.4	26	54.2	

Potential comorbidities	Hypertension	22	45.8	23	47.9	0.984
	Cerebrovascular disease	2	4.2	2	4.2	
	Coronary disease	7	14.6	5	10.4	
	Diabetes	10	20.8	11	22.9	
	Pulmonary insufficiency	7	14.6	7	14.6	

^*∗*^ *P* value ≤0.05 is significant.

**Table 2:** Operative data and technical aspect according to the surgical approach.

	Surgical approach				*P* value^*∗*^
	Open surgery		Laparoscopic		
	Mean	SD	Mean	SD	
Length of the surgical specimen (cm)	27.33	3.22	20.10	1.97	<0.001
Tumor size (cm)	5.63	.914	4.50	0.715	<0.001
Area of mesentery (mm^**2**^)	15.36	1.24	14.22	0.91	<0.001
Proximal resection margin (cm)	20.27	8.821	16.58	5.870	0.018
Distal resection margin (cm)	13.44	1.809	14.83	1.894	<0.001
Incision length (cm)	18.79	3.18	6.10	0.93	<0.001
Operative time (min)	152.04	27.24	201.31	57.80	<0.001
Blood loss (mL)	264.17	67.70	189.33	74.22	<0.001
No. of LNs retrieved	32.65	12.28	39.81	16.74	0.010
Distance from the tumor to the high tie (mm)	124.44	18.61	140.42	16.78	<0.001
Distance from the nearest bowel wall to the high tie (mm)	79.96	8.84	110.71	12.36	<0.001

^*∗*^ *P* value ≤0.05 is significant.

**Table 3:** Postoperative data according to the surgical approach.

	Surgical approach				*P* value^*∗*^
	Open surgery		Laparoscopic		
	Mean	SD	Mean	SD	
Pain scores	5.31	0.88	3.25	0.76	<0.001
Getting-out-of-bed time (days)	3.42	0.94	1.67	0.66	<0.001
First passage of flatus (hours)	100.48	11.63	56.77	9.51	<0.001
Time to resume liquid diet (day)	4.91	1.19	3.50	1.09	<0.001
Time of normal diet (day)	6.27	0.96	4.73	0.76	<0.001
Duration of hospital stay (day)	13.04	3.07	9.13	1.57	<0.001

^*∗*^ *P* value ≤0.05 is significant.

**Table 4:** Postoperative complications.

		Surgical approach				*P* value^*∗*^
		Open surgery		Laparoscopic		
		Count	%	Count	%	
Wound infection	No	44	91.7	47	97.9	0.362
	Yes	4	8.3	1	2.1	
Lymphatic leakage	No	47	97.9	47	97.9	1.00
	Yes	1	2.1	1	2.1	
Anastomotic leakage	No	46	95.8	47	97.9	0.557
	Yes	2	4.2	1	2.1	
Pulmonary infection	No	44	91.7	47	97.9	0.168
	Yes	4	8.3	1	2.1	
Ileus	No	40	83.3	46	95.8	0.04^*∗*^
	Yes	8	16.7	2	4.2	
Deep venous thrombosis	No	45	93.8	47	97.9	0.307
	Yes	3	6.3	1	2.1	
30-day mortality	No	47	97.9	47	97.9	1.00
	Yes	1	2.1	1	2.1	
Conversion rate	No	—	—	44	91.7	NA
	Yes	—	—	4	8.3	

^*∗*^ *P* value ≤0.05 is significant.

**Table 5:** Recurrence (local and distant) according to the surgical approach.

		Surgical approach				*P* value
		Open surgery		Laparoscopic		
		Count	%	Count	%	
Local recurrence	No	46	95.8	46	95.8	1.00
	Yes	2	4.2	2	4.2	
Distant metastasis	No	44	91.7	45	93.8	0.69
	Yes	4	8.3	3	6.3	

## Data Availability

The dataset used to support the findings of this study is available from the corresponding author upon request.
